# Presumable role of outer membrane proteins of *Salmonella* containing sialylated lipopolysaccharides serovar Ngozi, sv. Isaszeg and subspecies *arizonae* in determining susceptibility to human serum

**DOI:** 10.1186/s13099-015-0066-0

**Published:** 2015-07-16

**Authors:** Bożena Futoma-Kołoch, Urszula Godlewska, Katarzyna Guz-Regner, Agata Dorotkiewicz-Jach, Elżbieta Klausa, Jacek Rybka, Gabriela Bugla-Płoskońska

**Affiliations:** Department of Microbiology, Institute of Genetics and Microbiology, University of Wrocław, Przybyszewskiego 63-77, 51-148 Wrocław, Poland; Department of Pathogens’ Biology and Immunology, Institute of Genetics and Microbiology, University of Wrocław, Przybyszewskiego 63-77, 51-148 Wrocław, Poland; Regional Centre of Transfusion Medicine and Blood Bank, Czerwonego Krzyża 5, 50-345 Wrocław, Poland; Ludwik Hirszfeld Institute of Immunology and Experimental Therapy, Polish Academy of Sciences, Weigla 12, 53-114 Wrocław, Poland

**Keywords:** *Salmonella* O48, Lipopolysaccharide, Sialic acid, Complement, C3, Outer membrane protein

## Abstract

**Background:**

The O48 group comprises *Salmonella* bacteria containing sialic acid in the lipopolysaccharide (LPS). Bacteria with sialylated surface structures are described as pathogens that avoid immunological response of the host by making similar their surface antigens to the host’s tissues (molecular mimicry). It is known that the smooth-type LPS of *Salmonella enterica* and outer membrane proteins (OMP) PgtE, PagC and Rck mediate serum resistant phenotype by affecting complement system (C). The aim of this study was to investigate C3 component activation by *Salmonella* O48 LPS and OMP.

**Findings:**

In the present study, we examined C3 component deposition on the three *Salmonella* O48 strains: *S. enterica* subspecies *enterica* serovar Ngozi, *S. enterica* subsp. *enterica* sv. Isaszeg, and *S.enterica* subsp. *arizonae* containing sialic acid in the O-specific part of LPS. The greatest C3 deposition occurred on *Salmonella* sv. Isaszeg cells (*p* < 0.005) as well as on their LPS (low content of sialic acid in LPS) (*p* < 0.05) after 45 min of incubation in 50% human serum. Weaker C3 deposition ratio on the *Salmonella* sv. Ngozi (high content of sialic acid in LPS) and *Salmonella* subsp. *arizonae* (high content of sialic acid in LPS) cells correlated with the lower C3 activation on their LPS. Immunoblotting revealed that OMP isolated from the tested strains also bound C3 protein fragments.

**Conclusions:**

We suggest that activation of C3 serum protein is dependent on the sialic acid contents in the LPS as well as on the presence of OMP in the range of molecular masses of 35–48 kDa.

## Background

Despite the tremendous knowledge about the virulence factors of *Salmonella* bacteria, *Salmonella enterica* is still an important cause of gastrointestinal infections as well as systemic infections [1]. The mechanisms employed by *Salmonella* strains to evade host immunological defenses are not entirely understood. When the bacteria leave the intestinal tract, they may spread into the bloodstream where they are recognized by the complement system (C). System C is activated via three pathways: the alternative pathway (AP), the classical pathway (CP), and the lectin pathway (LP). The pathways converge at the step of C3 deposition, the crucial stage in C activation [2].

Incorporation of the sialic acid into the bacterial surface glycoconjugates usually results in an increase of serum resistance (SR) of some Gram-negative bacteria such as *Neisseria meningitidis*, *Neisseria gonorrhoeae*, *Haemophilus somnus* [3–5]. Our previous investigations showed that the presence of sialylated LPS in a group of *Salmonella* O48 (21 strains) was not sufficient to relate bacterial resistance (SR) to the bactericidal activity of human serum (HS), and bovine serum (BS).

Nothing is known about the influence of sialylated bacterial surface structures on C3 fixation in serum. The role of the outer membrane proteins (OMP) in *Salmonella* susceptibility to serum has not also been investigated fully. It is known that surface-exposed protein PagC (17–19 kDa) confers SR in *Salmonella* sv. Choleraesuis (O:7) [6] and Rck (17 kDa) of *Salmonella* sv. Typhimurium (O:4) and Enteritidis (O:9) expressed in *Escherichia coli* BL21 were associated with resistance to AP [7]. Ramu et al. [8] described an example of a surface protein PgtE (35 kDa) of *S. enterica* that proteolytically cleaved C3 and enhanced bacterial resistance to human serum. This paper highlights the importance of sialylated LPS and OMP in C3 protein binding.

## Methods

### Bacterial strains

**Figure 1:**
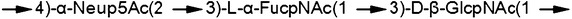
The structure of the O-antigen from lipopolysaccharide of *Salmonella* *enterica* species serotype of O48. *Neup5Ac* *N*-acetylneuraminic acid, sialic acid.

Bacteria belonging to the serogroup of O48 are characterized by the presence of a smooth type LPS containing sialic acid (Neup5Ac, *N*-acetylneuraminic acid) (Figure 1) [9]. Three laboratory strains were used: *S. enterica* subspecies *enterica* serovar Ngozi (PCM 2514), *S. enterica* subsp. *enterica* sv. Isaszeg (PCM 2550), *S.enterica* subsp. *arizonae* (PCM 2543) (PCM—Polish Collection of Microorganisms) (Table 1).

**Table 1:** The origin, antigenic and phenotypic characteristic of the *Salmonella* O48 strains used in this study

*Salmonella enterica* subspecies	Serotype	Group O48 antigen H	Source	Sialic acid/Kdo ratio (%) in LPS^a^	C activation pathway in human serum^a^
*Salamae*	Ngozi	z_10_: [1, 5]	PCM^b^ 2514 KOS^c^ 1250	239	CP/LP + AP^f^
*Arizonae*	–	z_36_: –	PCM 2543 CNCTC^d^ Ar 4/50	200	AP^g^
*Enterica*	Isaszeg	z_10_: e, n, x	PCM 2550 IP^e^ 886/71	<1	CP/LP/AP^h^

*CP* classical pathway of complement system (C), *LP* lectin pathway of C, *AP* alternative pathway of C.

^a^[16].

^b^PCM—Polish Collection of Microorganisms, Ludwik Hirszfeld Institute of Immunology and Experimental Therapy, Polish Academy of Sciences, Wroclaw, Poland.

^c^KOS National *Salmonella* Center, Gdansk, Poland.

^d^CNCTC Czech National Collection of Type Cultures, National Institute of Public Health, Prague, Czech Republic.

^e^IP Institute Pasteur, Paris, France.

^f^Parallel activation of the CP/LP pathways and AP in the bactericidal mechanism of C activation.

^g^Activation of AP.

^h^Independent activation of the CP/LP pathways or AP in the bactericidal mechanism of C activation.

### Sera

Human serum (HS) was taken from 20 healthy donors in Regional Centre of Transfusion Medicine and Blood Bank the name of Prof. T. Dorobisz in Wrocław, Poland. It was conducted according to the principles expressed in the Declaration of Helsinki. The serum was frozen in 1 ml aliquots at −70°C for a period no longer than 3 months. A suitable volume of serum was thawed immediately before use. Each portion was used only once.

### Determination of the C3 concentration in human serum (HS)

The concentration of C3 in HS was estimated with radial immunodiffusion method using specific antibodies (The Binding Site).

### Bactericidal assay

The bactericidal assay was performed as described previously [10]. Briefly, log-phase cultures of the bacteria were suspended in 50% human serum (HS) or 50% heat inactivated serum (56°C for 30 min, HS-IN) and incubated at 37°C with the numbers of viable *Salmonellae* determined by serial dilution and plating in triplicate on Luria–Bertani agar at various time points. The number of colony-forming units (CFU/ml) at time 0 was taken as 100%. Strains with survival rates below 90% after 45 min of incubation were classified as serum sensitive (SS).

### Isolation of outer membrane proteins (OMP) and lipopolysaccharide (LPS)

The isolation of OMP from the tested strains were performed with the detergent Zwittergent Z 3–14^®^ (Calbiochem) according to Murphy and Bartos [11]. OMP quantifications were done with a bicinchoninic acid (BCA) Protein Assay Kit (Pierce). LPS was isolated with an LPS Extraction Kit according to the manufacturer’s instruction (Intron Biotechnology).

### Enzyme-linked immunosorbent assay (ELISA)

Complement C3 binding assay for bacterial cells was carried out by indirect ELISA according to Alberti et al. [12]. Complement C3 activation assay for LPS was performed by a direct sandwich ELISA according to Holmskov-Nielsen [13]. The assays were performed on the pool of HS with the correct of parameters of C3 complement protein and lacking specific anti-*Salmonella* antibodies. Polyclonal rabbit anti-human C3c (Dako) (2.5 μg/ml), and polyclonal goat anti-rabbit immunoglobulins/HRP (horseradish peroxidase-conjugated, Dako) were used to detect C3 activated fragments. The plates were developed with *O*-phenylenediamine dihydrochloride (SIGMAFAST™ OPD, Sigma-Aldrich) and measured at A_492_.

### Electrophoresis and immunobloting

Samples of OMP complexes were electrophoresed under reducing conditions (SDS–PAGE) on 6 and 12.5% gels by the method of Laemmli [14] and in native (nonreducing) conditions (blue native polyacrylamide gel electrophoresis, BN-PAGE) on 2 and 12% gels according to Swamy et al. [15]. The samples were then transferred to PVDF membranes and immunoblotted to detect C3 fragments bound to OMP. Western Blot Signal Enhancer (Pierce) was used before blocking nonspecific binding sites on the PVDF membrane. Coomassie brilliant blue staining demonstrated protein band patterns characteristic of tested *Salmonellae* OMP complexes. The detection of C3 bound to OMP was done with polyclonal rabbit anti-C3c antibodies (Dako) diluted in the proportion of 1/400 and the polyclonal goat anti-rabbit immunoglobulins/HRP (Dako) diluted 1/2000. Blots were imagined with an Opti-4CN Substrate Kit (Bio-Rad). The results were confirmed in three independent experiments.

### Statistical analysis

The data obtained for the bactericidal activity of HS were statistically analyzed using the ANOVA Kruskal–Wallis test. Comparisons between the strains and their LPS in the ELISA tests were made with the ANOVA Friedman and Kendall rank correlation coefficient test (Statistica.pl v. 9.0, Statsoft, Krakow, Poland).

## Results

**Figure 2:**
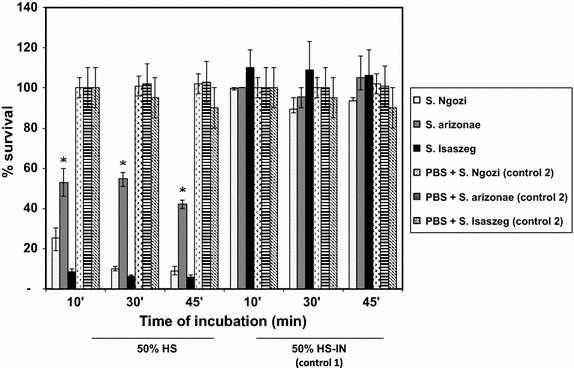
Susceptibility of *Salmonella* strains to the antibacterial activity of human serum (HS). Log-phase cultures of the bacteria (1 × 10^5^ CFU/ml) were incubated in 50% human serum (HS), in 50% heat inactivated serum (56°C for 30 min, HS-IN, control 1) or PBS (control 2) for 45 min. Serial dilutions were performed to calculate colony forming units (CFU/ml). The average number of colonies was estimated from three plates. The CFU/ml at time 0 was taken as 100%. Sensitivity to HS differs significantly if *p* values are less than 0.05 (*).

**Figure 3:**
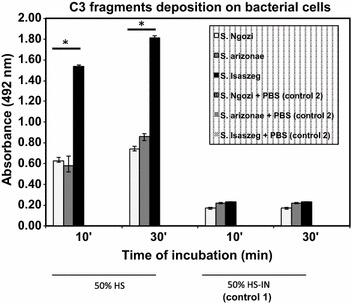
C3 complement protein depositions on the bacterial cells. Indirect enzyme-linked immunosorbent assay (ELISA). Bacterial cells in log-phase (1 × 10^7^ CFU/ml) were incubated in 50% HS, 50% HS-IN (control 1) or PBS (control 2) for 30 min at 37°C. Activation of C3 differs significantly if the *p* values are less than 0.005 (*).

The concentration of C3 in HS pool was at the level of 1,217 mg/l. The reference concentration in healthy human serum is in the range of 970–1,576 mg/l. Three tested strains were SS (Figure 2). There was significant difference in the killing of the tested strains. The greatest decrease in bacterial numbers was observed for *Salmonella* sv. Ngozi and *Salmonella* sv. Isaszeg. *Salmonella* subsp. *arizonae* turned out to be the less sensitive to the bactericidal activity of HS (*p* < 0.05). Its survival rate reached 53% after 10 min of incubation, 55% after 30 min of incubation, and 42% after 45 min of incubation. HS-IN was performed in order to confirm that killing of bacteria was C-mediated. Thus, their survival was not reduced significantly. Having the knowledge about the susceptibility of sialylated *Salmonella* strains to HS, the extent of C3 complement components deposition on the bacterial cells was determined (Figure 3). The highest C3 deposition rate was noted for *Salmonella* sv. Isaszeg (*p* < 0.005). In control 1 (HS-IN), an A_492_ value was measured about 0.3, for control 2 no A_492_ signal was detected.

**Figure 4:**
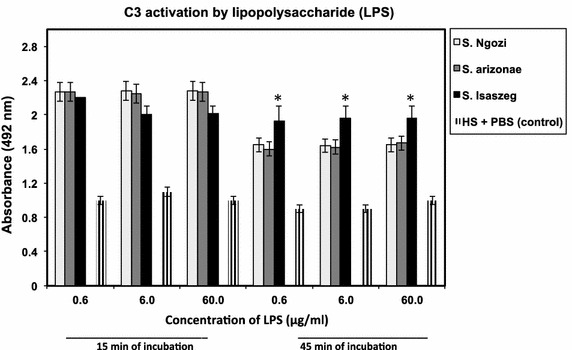
C3 complement protein depositions on immobilized LPS. Direct Sandwich ELISA. Microtiter plate wells were coated for 2 h at 37**°**C with polyclonal rabbit anti-C3c diluted 1/500 in 0.1 M sodium carbonate buffer (pH 9.6). Mixtures of LPS (0.6; 6.0; 60.0 μg/ml) and 80% HS were incubated for 45 min at 37**°**C. Activation of C3 differs significantly if the *p* values are less than p < 0.05 (*).

**Figure 5:**
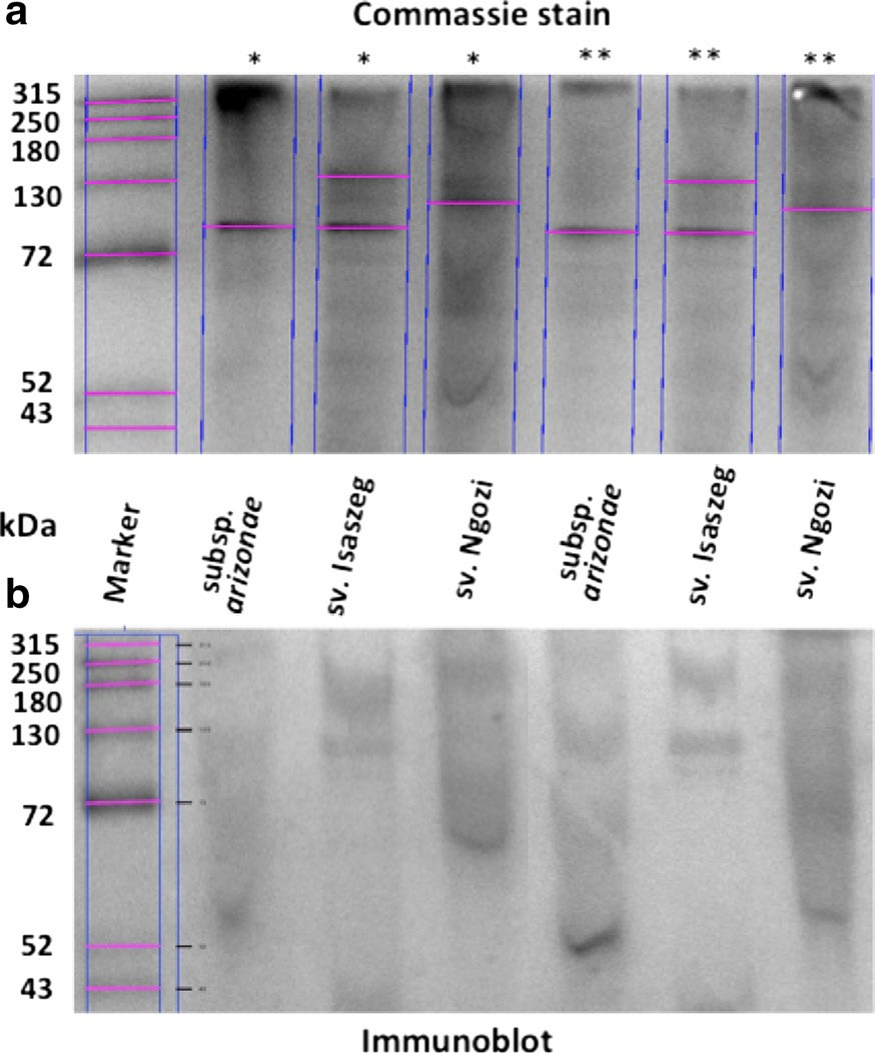
Immunoblot detection of C3c fragments on native OMP. OMP isolated with Zwittergent Z 3–14 detergent^®^. OMP patterns were determined by blue native polyacrylamide gel electrophoresis (BN-PAGE) (**a**) and C3 binding confirmed by Western blotting (**b**). Electrotransfer conducted at 100 V for 1 h. *Lane 1* molecular-weight marker 26625 (Thermo Scientific). The OMP concentrations were 10 μg/well (*), and 12 μg/well (**), respectively.

**Figure 6:**
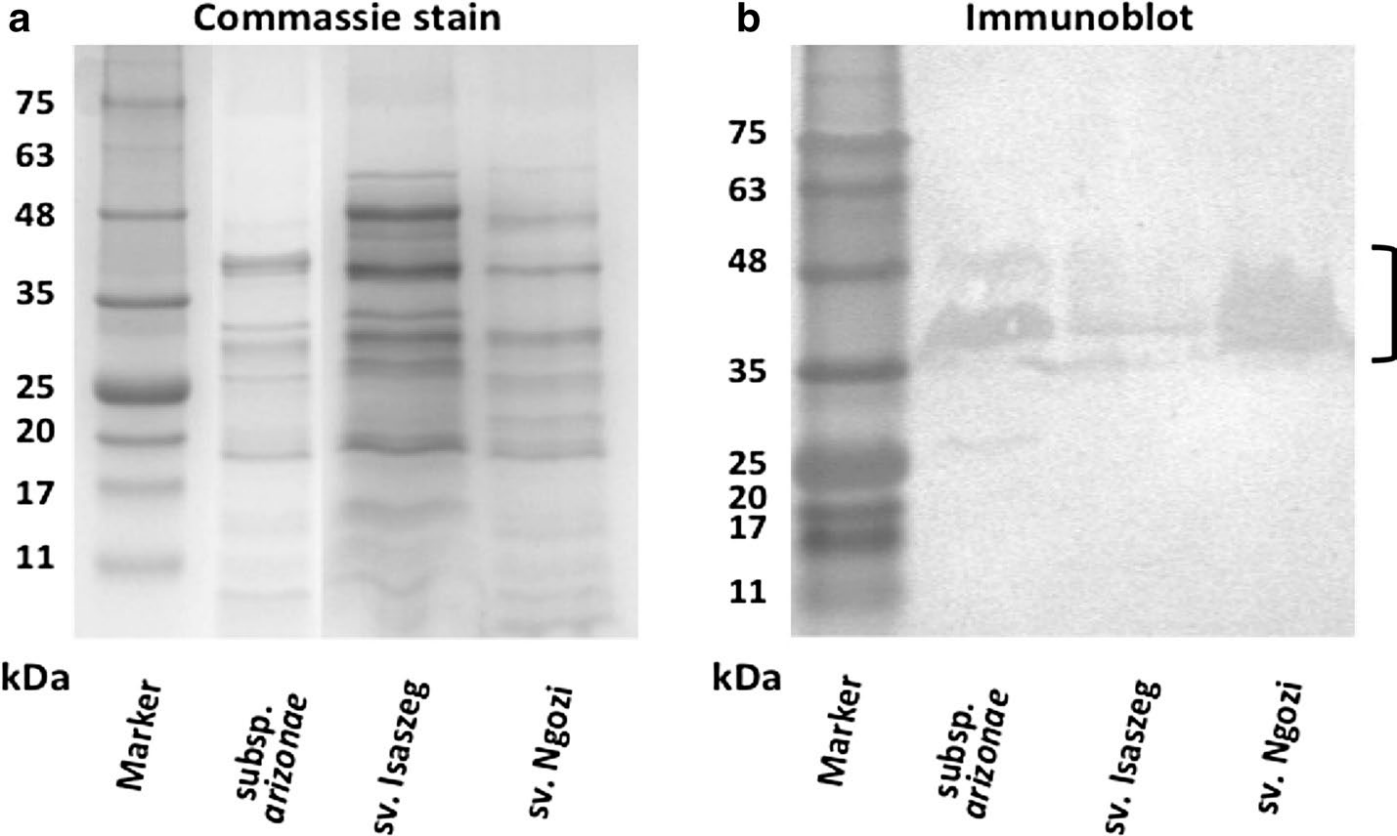
Immunoblot detection of C3c fragments on outer membrane proteins (OMP) under reducing conditions. OMP isolated with Zwittergent Z 3–14 detergent^®^ OMP patterns were determined by sodium dodecyl suphate-polyacrylamide gel electrophoresis (SDS–PAGE) (10 μg/well) (**a**) and C3 binding confirmed by Western blotting, (20 μg/well) (**b**). Electrotransfer conducted at 50 V for 1 h. *Lane 1* molecular-weight marker A8889 (AppliChem).

The results obtained for the surface antigens indicated that both LPS (direct sandwich ELISA) and OMP (immunoblots) antigens bound C3. It was observed C3 component activation occurred at a similar rate for LPS isolated from *Salmonella* at 15 min (*p* > 0.05) but different after 45 min of incubation (Figure 4) with the highest A_492_ for *Salmonella* sv. Isaszeg (*p* < 0.05). The low absorbance (A_492_ = 0.4) was noted for control sets containing HS or PBS. Considering OMP, C3 binding was obtained when OMP were electrophoresed in nonreducing conditions (BN-PAGE) (Figure 5). SDS–PAGE produced additional information that efficient C3 fixation occurred on the OMP bands of the molecular masses in the range of 35–48 kDa (Figure 6).

## Discussion

*Salmonella* O48 is the only group possessing sialylated LPS among all known *Salmonella* bacteria. In this study, we investigated the role of LPS and OMP in determining the susceptibility of three O48 strains: *Salmonella* sv. Isaszeg, *Salmonella* subsp*. arizonae*, and *Salmonella* sv. Ngozi to HS. We have confirmed our previous findings that the presence of smooth type, sialylated LPS in *Salmonella* O48 isolates did not protect against HS C-mediated killing [16] (Table 1), in contrast to others’ previous reports [5, 17, 18]. Sialylated lipopolysaccharide (LPS) was described in the context of molecular mimicry phenomenon connected to the onset of autoimmunity in humans [19]. Most autoimmune diseases are chronic, and they may be amplified by past infections [20].

The novelty of this paper is the first analysis of C3 activation on *Salmonella* isolates belonging to the O48 serogroup and their surface antigens: LPS and OMP. In general, endotoxin is the strong stimulant for the immune system, but in this case, the sialic acid substitutions in the O-specific region of LPS (Table 1) of *Salmonella* subsp*. arizonae* [sialic acid/Kdo ratio (%) = 200], and *Salmonella* sv. Ngozi [sialic acid/Kdo ratio (%) = 239] seemed to inhibited C3 activation in HS (Figure 4). *Salmonella* sv. Isaszeg LPS, which was characterized to contain sialic acid at almost non-detectable level [sialic acid/Kdo ratio (%) < 1], enhanced the C3 protein fixation. C3 protein binding to *Salmonella* sv. Isaszeg LPS was more constant, while in the case of *Salmonella* subsp*. arizonae* and *Salmonella* sv. Ngozi seemed to decay over time. The greatest C3 binding on the *Salmonella* sv. Isaszeg LPS might also reflect the higher C3 fragmentation on the bacterial cells.

It was demonstrated that OMP also interact with the C3 complement component. Western blotting in which OMP under reducing condition were used helped to show that C3 binding occurs on the OMP in the range of molecular masses of 35–48 kDa. It is worth emphasizing that this paper describes a method of immunoblotting performed for OMP isolated with a zwitterionic detergent. It is methodologically easier to show C3 binding onto the native OMP (BN-PAGE) but the weakness of this technique is a poor resolution of the OMP, hence it is not possible to point out distinct OMP, which interact with C3 fragments. In turn, SDS–PAGE is useful to resolve proteins as distinct bands, but the electrotransfer occurs less efficiently, therefore the signals coming from the detected C3 fragments on OMP are less satisfactory.

Our results relate to a problem of molecular mimicry in which the microorganisms’ camouflage occurs through sialic acid incorporation into bacterial surface structures. The phenomenon of mimicry as a cause of autoimmune diseases is unknown, and its reason has been discussed [21, 22]. In the future, the premises like those presented in this paper may be helpful to construct antibacterial vaccines against autoimmune-connected infections.

## Conclusions

We might conclude, that both sialic acid containing LPS and OMP of *Salmonella* O48 play important role in C3 activation. We suggest that the differential sensitivity of tested bacteria to HS may be due to a weaker C3 activation on strongly sialylated LPS and a binding of C3 components to the OMP in the range of molecular weights of 35–48 kDa.
